# RETRACTION: Effect of Radiation Therapy During Surgery on Postoperative Wound Complications after Breast Reconstruction in Patients With Breast Cancer: A Meta‐Analysis

**DOI:** 10.1111/iwj.70367

**Published:** 2025-03-17

## Abstract

**RETRACTION**: HuangN. , LiuL. , QinY. , and XieY. , “Effect of Radiation Therapy During Surgery on Postoperative Wound Complications after Breast Reconstruction in Patients With Breast Cancer: A Meta‐Analysis,” International Wound Journal 21, no. 2 (2024): e14473, 10.1111/iwj.14473.PMC1082852837905575

The above article, published online on 31 October 2023, in Wiley Online Library (http://onlinelibrary.wiley.com/), has been retracted by agreement between the journal Editor in Chief, Professor Keith Harding; and John Wiley & Sons Ltd. Following an investigation by the publisher, all parties have concluded that this article was accepted solely on the basis of a compromised peer review process. The editors have therefore decided to retract the article. The authors did not indicate their agreement with the retraction.

**RETRACTION**: N. Huang , L. Liu , Y. Qin , and Y. Xie , “Effect of Radiation Therapy During Surgery on Postoperative Wound Complications after Breast Reconstruction in Patients With Breast Cancer: A Meta‐Analysis,” International Wound Journal 21, no. 2 (2024): e14473, 10.1111/iwj.14473.PMC1082852837905575

The above article, published online on 31 October 2023, in Wiley Online Library (http://onlinelibrary.wiley.com/), has been retracted by agreement between the journal Editor in Chief, Professor Keith Harding; and John Wiley & Sons Ltd. Following an investigation by the publisher, all parties have concluded that this article was accepted solely on the basis of a compromised peer review process. The editors have therefore decided to retract the article. The authors did not indicate their agreement with the retraction.

